# Fitness of evolving bacterial populations is contingent on deep and shallow history but only shallow history creates predictable patterns

**DOI:** 10.1098/rspb.2022.1292

**Published:** 2022-09-14

**Authors:** Chelsea E. Smith, Adam N. H. Smith, Tim F. Cooper, Francisco B.-G. Moore

**Affiliations:** ^1^ Department of Biological Sciences, Kent State University, Kent, OH 44242, USA; ^2^ School of Mathematical and Computational Sciences, Massey University, Auckland 0634, New Zealand; ^3^ School of Natural Sciences, Massey University, Auckland 0634, New Zealand; ^4^ Department of Biology, University of Akron, Akron, OH 44325, USA

**Keywords:** contingency, epistasis, adaptation, experimental evolution, genotype-by-environment interaction

## Abstract

Long-term evolution experiments have tested the importance of genetic and environmental factors in influencing evolutionary outcomes. Differences in phylogenetic history, recent adaptation to distinct environments and chance events, all influence the fitness of a population. However, the interplay of these factors on a population's evolutionary potential remains relatively unexplored. We tracked the outcome of 2000 generations of evolution of four natural isolates of *Escherichia coli* bacteria that were engineered to also create differences in shallow history by adding previously identified mutations selected in a separate long-term experiment. Replicate populations started from each progenitor evolved in four environments. We found that deep and shallow phylogenetic histories both contributed significantly to differences in evolved fitness, though by different amounts in different selection environments. With one exception, chance effects were not significant. Whereas the effect of deep history did not follow any detectable pattern, effects of shallow history followed a pattern of diminishing returns whereby fitter ancestors had smaller fitness increases. These results are consistent with adaptive evolution being contingent on the interaction of several evolutionary forces but demonstrate that the nature of these interactions is not fixed and may not be predictable even when the role of chance is small.

## 1. Introduction

Evolutionary outcomes are determined by core factors that shape the diversity of life: adaptation, chance and history [1]. Adaptation reflects the power of natural selection to drive populations along evolutionary paths to phenotypes of high fitness. If few paths are available, replicate populations will follow repeatable, perhaps even predictable, outcomes [2–5]. By contrast, chance and history promote evolutionary divergence. Chance causes divergence between populations through stochastic differences in the occurrence and success of newly arising mutations [6–9]. History, defined here as differences in the genetic starting points of selected populations, promotes divergence if evolutionary opportunities or constraints are contingent on specific genotypes [10–15]. Determining the relative contribution of these forces, and how this might depend on the selective environment, is crucial to the goal of predicting evolutionary outcomes.

Chance events are an unavoidable part of evolution. Nevertheless, the effect of chance may be overwhelmed if there are only a limited number of available beneficial changes, leaving adaptation as the dominant force in determining evolutionary outcomes. In this view, though there may be distinct routes to an evolutionary outcome, different paths will tend to converge [16–18]. Examples include the independent evolution of functionally similar eyes [19], toxins [20] and electric organs [21]. Alternatively, genetic interactions—epistasis—that cause the selective benefit of mutations to depend on the genetic background on which they occur can amplify the effect of mutational differences [3,15,22–26]. These interactions can lead to repeatably different evolutionary outcomes depending on a starting genotype when they depend on mutations that are distinct between those genotypes. They can also lead to divergence between replicate populations started from the same genotype when they depend on new mutations arising during evolution. Together, the effects of chance and history make the evolutionary dynamic much more complex than a repeatable process of optimization.

The contributions of adaptation, chance and history to evolution have largely been investigated by examining populations that have evolved under similar selective pressures [1]. For example, independent populations of Anolis lizards have converged on similarly selected morphologies [27]. However, even as similar selection pressures lead to similar outcomes, populations with distinct genetic starting points have evolved distinct phenotypes [28]. The complication of initial differences between evolving populations can be controlled for in laboratory experiments that start with genetically identical replicate populations. Analysis of a long-term experiment evolving initially identical replicate populations of *Escherichia coli* found similar overall changes in fitness, though with significant and sustained variation [29,30]. Moreover, one population evolved a novel phenotype via an evolutionary path that depended on a series of earlier mutations [10,31,32]. Other studies have found divergence among replicated populations dependent on the selective environment [33–35].

Laboratory studies that have tracked the evolution of populations starting with distinct genotypes have typically found that this history had a significant influence on evolutionary outcomes [11,15,25,26,36–44]. Of note, a significant proportion of this effect often depends on the starting fitness of a population, following a pattern of diminishing returns whereby fitter populations adapt more slowly than less fit populations [25,26,41,45,46]. Several studies have also identified genotypes that have significant differences in their evolutionary potentials that are not determined by initial fitness [11,36,47]. Indeed, analyses of mutational pathways have sometimes discovered intermediate genotypes crucial to determining future evolutionary outcomes [31,48,49].

A common limitation of studies examining the role of history on evolutionary outcomes is a focus on closely related populations that differ only by mutations acquired during short periods of laboratory adaptation [25,26,43]. High relatedness of starting populations probably reduces the potential influence of history on evolution because newly arising mutations will be tested on similar genetic backgrounds. The more diverse genetic backgrounds of individuals isolated from natural populations increases the chance that they will explore different regions of a fitness landscape during evolution, leading to divergent outcomes [11]. Analyses of evolving populations founded from individuals sampled directly from natural populations have found significant effects of history, though this tends to be weaker for the traits most closely related to fitness [50]. To our knowledge, however, no study has compared the contribution of historical effects at different levels of divergence, as is needed to assess how rapidly history can redirect evolutionary outcomes.

**Figure 1.**
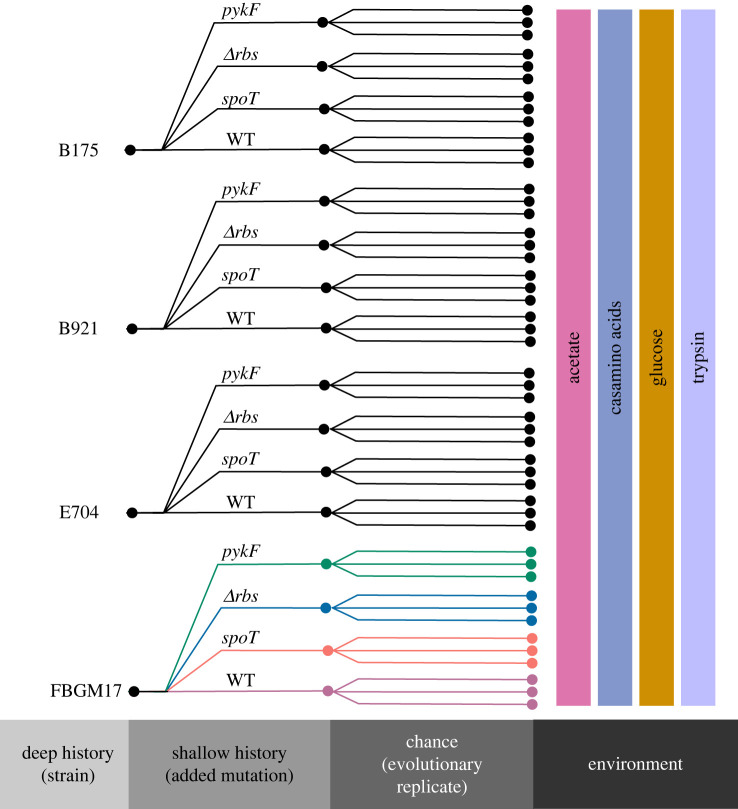
Schematic of evolved population organization. Four progenitor natural isolate strains of *E. coli* (deep history) were each divided into four sub-groups comprising the original strain (wild type (WT)) and three new strains with distinct added mutations that were selected in populations evolved as part of an earlier experiment (shallow history) [24,51]. Each of the 16 strains was used to found three replicate populations (chance) in each of four selection environments. Each population was evolved for 2000 generations and then fitness changes compared against its progenitor. (Online version in colour.)

In this study, we compared the roles of adaptation, chance and history on the evolution of populations of *E. coli*. Four natural isolate strains of *E. coli* (deep history) were each engineered with three beneficial mutations (shallow history). Each strain–mutation combination was evolved with threefold replication (chance) for 2000 generations in four resource environments (figure 1). This crossed and replicated design allowed us to partition the variation in evolutionary outcomes into the effects of deep and shallow history, adaptation and chance.

## 2. Material and methods

### (a) Bacterial strains and media

In a previous study, 26 natural isolate strains of *E. coli* were isolated and engineered by adding between one and four mutations [52]. We chose four of these natural isolates as progenitor strains (E704, B921, B175 and FBGM17; representing ‘deep history’) that each had three mutations (representing ‘shallow history’) separately added to comprise a set of 16 founder strains (figure 1). Mutations were added in the following genes or gene regions: *spot, pykF* and *rbs*. These mutations occurred early in the evolution of an *E. coli* population selected in minimal medium supplemented with glucose and conferred a significant fitness benefit in the background in which they arose [24,51]. At least the *spoT* and *pykF* mutations are likely to be highly pleiotropic, impacting core regulatory (*spoT*) or metabolic (*pykF*) processes, and therefore are good candidates for interacting with newly arising mutations to influence available evolutionary outcomes [23,53].

Populations started with founder strains were propagated in Davis-Mingioli minimal medium supplemented with glucose (0.5 g l^−1^), casamino acids (1.4 g l^−1^), acetate (0.36 g l^−1^) or trypsin (0.064 g l^−1^). These four resource environments were chosen following previous work [46,51,54]. Glucose was chosen as a reference because it is a preferred resource for *E. coli* frequently used in other long-term evolution studies [46,51]. Casamino acids are a mixture of amino acids that provides a rich growth environment. Acetate is a weak acid that is a by-product of *E. coli* growth in glucose [55]. Trypsin is a protein that must be degraded prior to uptake and was expected to impose a stressful low-nutrient environment.

### (b) Experimental design

We carried out a long-term evolution experiment to investigate the relative contribution of adaptation, deep and shallow history, chance and selection environment, on the evolution of fitness. Populations were founded with each of the 16 strains described above, giving a crossed design of shallow history (added mutation) by deep history (progenitor natural isolate strain). Each strain was used to start 12 replicate populations, with three of these evolved in each of the four different resource environments, giving a total of 192 evolving populations (figure 1). Each population was propagated at 37°C for 2000 generations in unshaken test tubes with daily transfer of 5 µl to 5 ml of fresh medium.

### (c) Competitive fitness assays

We performed one-day competitive fitness assays to quantify the relative fitness of evolved strains. The 16 founder strains we used were able to grow on arabinose (Ara+) and otherwise isogenic Ara- derivatives were isolated previously [52]. These two sets of strains can be distinguished visually on tetrazolium arabinose (TA) indicator agar [51]. In competitions between reference Ara- and evolved Ara+ strains, samples were grown from frozen stocks for 24 h in lysogeny broth (LB) and then transferred to allow growth in their respective evolution resource environment for an additional 24 h. After this preconditioning, competitions were started by mixing equal volumes of an evolved population and its corresponding Ara- ancestor at a combined 1000-fold dilution in fresh media. A sample was immediately plated on TA agar to determine the initial frequency of each competitor. A second sample was plated following 24 h of competition. Malthusian parameters of each competitor were estimated as: *m* = ln(*N*_1_/*N*_0_), where *N*_0_ and *N*_1_ represent initial and final densities, respectively. The relative fitness of evolved populations was calculated as the ratio of Malthusian parameters of evolved and ancestral competitors. The effect of the Ara marker in each strain–resource combination, as determined by control competitions between Ara+ and Ara- ancestor strains, was subtracted from relevant fitness estimates. Competition assays were carried out in complete blocks with fourfold replication.

### (d) Estimation of natural isolate strain fitness using growth curves

Competitive fitness assays allow fitness of derived or evolved strains to be compared to a corresponding progenitor strain. It is not possible to extend this approach to compare the fitness of our distinct progenitor strains because competition between these strains may be affected by complex interactions, for example, mediated by colicins or phage, that lead to non-transitive fitness relationships. To estimate fitness of all starting and evolved strains on a comparable scale, we analysed growth curves of progenitor strains to estimate their fitness relative to one another as determined only by differences in resource use [56]. In subsequent comparisons between progenitor strains, we chose E704 as the reference strain because it had the lowest mean fitness across the four assay environments. The fitness of progenitor strains estimated in each environment was added to competitive fitness estimates of evolved populations to obtain final fitness values for all evolved populations.

### (e) Genetic and biology relatedness

Core and accessory genomes were estimated by aligning 250 base windows across a panel of 90 natural isolate *E. coli* strains using PANSEQ [57]. PhyML was used to build a maximum-likelihood tree of the core genome. For the accessory genome, a binary input file indicating the presence/absence of each accessory gene in each strain was analysed using default parameters of PARS in PHYLIP [58]. The diet profiles of the strains were measured using Biolog PM1 plates. These plates allow estimation of the respiratory activity of each strain on each of 95 distinct substrates (Biolog, Hayward, CA). A neighbour-joining tree was constructed using Biolog data using the program Neighbor in PHYLIP [58]. To determine the fraction of common genes in the progenitor strains used in this study we annotated genomes using Prokka 1.14.6 [59] and compared them to identify core and accessory genes using Roary 3.11.2 with amino acid alignment [60].

### (f) Statistical analysis

We used mixed-effects linear models to partition variation in final fitness and the difference between initial and final fitness (change in fitness) among the various factors in the experimental design. Statistical analyses were performed using R 4.1.0 [61]. Estimation of variance components was performed using the lmer function from the lme4 package [62]. CIs of variance components were calculated using the confint function of the VCA package. We tested for an effect of initial fitness as a covariate by fitting models with and without that term and comparing them using the anova function. In all cases, the fit of models was improved by the addition of an assay block effect and this effect was included in models reported here. History was assessed at the level of progenitor strain (deep history) and of added mutation (shallow history) by adding these as random effects and estimating their s.d. as the square root of their respective variance components. Reporting the s.d. gives values of these effects comparable to the mean change in fitness due to adaptation. Chance was estimated similarly as the s.d. of the variance component of replicate populations nested under each founder strain. Adaptation was reported as the grand mean fitness or fitness improvement of relevant evolved populations.

## 3. Results

### (a) Influence of history, chance and environment on the fitness of evolved populations

**Figure 2.**
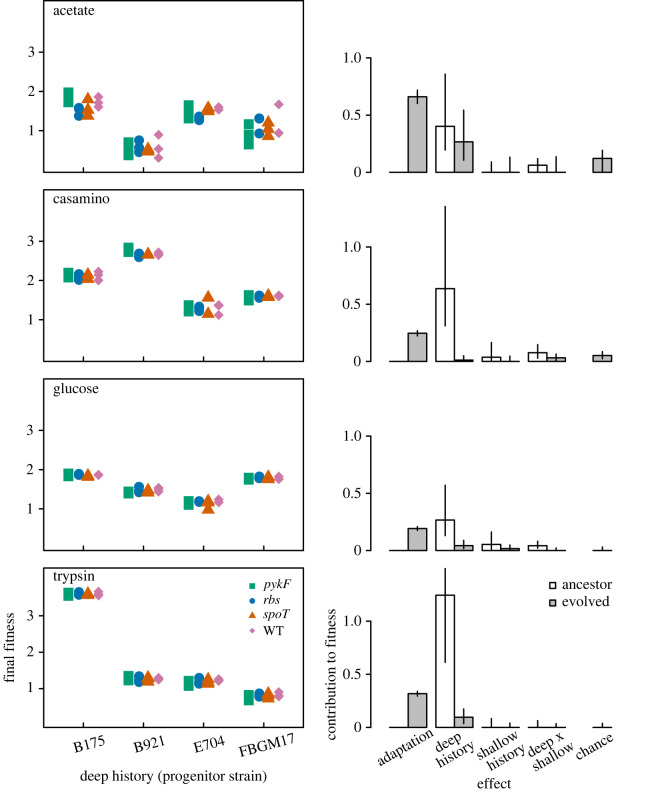
Contribution of adaptation, deep history, shallow history and chance to final fitness in each environment. Left-hand panels indicate the final fitness of replicate founder strain populations evolved in each environment. The contribution to fitness of adaptation, deep and shallow history, their interaction and chance, are indicated in the right-hand barplots. Contributions were estimated as the square root of the relevant variance components. Hollow and solid bars indicate ancestral versus evolved populations, respectively. Note we could not estimate effects of chance or adaptation for ancestral populations. Error bars indicate 95% CIs. All fitness estimates were performed with fourfold replication. (Online version in colour.)

We evolved four natural isolate strains and derivatives containing one of three additional mutations for 2000 generations in each of four selection environments (figure 1). After this evolution, we determined the relative influence of adaptation, deep (strain) and shallow (mutations added to each strain) history, the interaction between deep and shallow history and chance (variation among evolutionary replicates) on fitness (figure 2). Adaptation was significant in all environments, reflecting an increase in fitness at the end of the experiment. Deep history explained a significant amount of variation in fitness between strains at both the start and end of the experiment. Indeed, in the casamino acids and trypsin environments, deep history contributed more than adaptation to final population fitness. The effect of shallow history was initially small and changed little following evolution. Shallow history did, however, interact significantly with deep history in the casamino acids and glucose selection environments, indicating that introducing different engineered mutations resulted in different final fitness in different progenitor strain backgrounds. Finally, chance effects were generally small and not significant. Together, these results indicate a substantial influence of adaptation and an ongoing effect of deep history in determining population fitness after a period of lab evolution.

### (b) Effect of history on fitness change

**Figure 3.**
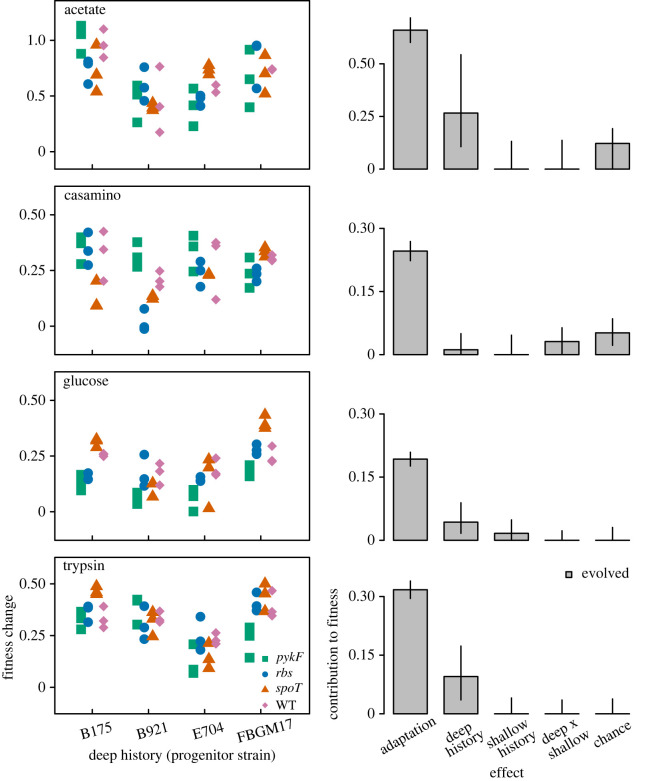
Contribution of deep history, shallow history and chance to change in fitness in each environment. Organization of plots are as for figure 2 except that effects are only considered for evolved populations because fitness change is not applicable to ancestral strains. Note different Y-axis scale for populations evolved in acetate. (Online version in colour.)

A complication of the above analysis is that initial fitness differences between progenitor strains are large relative to changes in fitness that occurred during the evolution experiment (electronic supplementary material, figure S1). For this reason, we repeated the analysis using fitness change relative to the relevant progenitor strain as the response variable, thereby removing the direct effect of initial fitness on determining final fitness. The importance of deep history was smaller in these models, though it remained significant in acetate, glucose and trypsin selection environments (figure 3). The effect of shallow history increased, becoming similar to that of deep history in the casamino acids and glucose environments, and interacting significantly with deep history in all but the acetate environment. Again, chance effects were generally small and not significant. These results indicate a dominance of adaptation in determining fitness changes but also that history continues to have a significant influence.

### (c) Initial fitness of strains predicts shallow, but not deep, history effects on fitness change

Previous studies have found that initial fitness can influence rates of adaptation, and it is possible that some of the variation in evolved fitness changes can be explained by the different fitness of founder strains, rather than on specific effects of their genotypes [25,26,41,52,63,64]. In particular, many studies have found a pattern of diminishing returns, whereby fitter populations tend to have lower rates of adaptation than less fit populations [25,26,41,45]. To test this, we repeated the above analysis of fitness change explicitly including the initial fitness of each founder strain as a covariate. We found that initial fitness had a significant, or marginally significant, effect on fitness change only for populations evolved in the casamino acids and glucose environments (electronic supplementary material, table S1, figure S2). Counter to our expectation, however, this relationship was generally positive, such that strains with high initial fitness had higher fitness increases (Pearson correlations: acetate: *r* = 0.13, *p* = 0.38; casamino: *r* = −0.34, *p* = 0.02; glucose: *r* = 0.27, *p* = 0.06; trypsin: *r* = 0.19, *p* = 0.20; electronic supplementary material, figure S3).

To test if the generally weak relationship between initial fitness and changes in fitness also held among the much more closely related strains derived from each natural isolate, i.e. at the level of shallow history, we repeated the above analysis separately for each progenitor natural isolate strain. Here, we found strong support for a pattern of diminishing returns: more fit derivatives of each progenitor improved less than less fit derivatives (Pearson correlations were negative in 15/16 comparisons, binomial test *p* < 0.001; electronic supplementary material, figure S3, table S2). These results indicate that initial fitness can predict the potential for fitness change among closely related strains that differ in their shallow history, but not among more distantly related strains.

### (d) Evolution environment contributes to fitness differences

**Figure 4.**
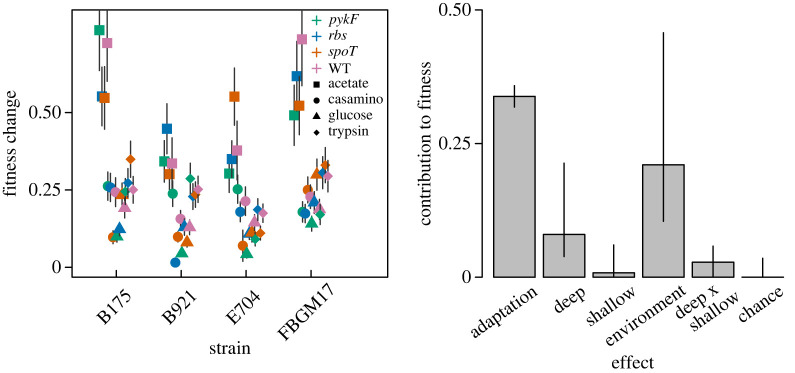
Dependence on environment of chance adaptation and history effects. Fitness change of evolved populations over all evolution environments. The contribution to fitness of deep and shallow history, evolution environment, the interaction between deep and shallow history, adaptation and chance, are indicated in the right-hand barplot. Error bars indicate 95% CIs.

To evaluate the dependence of selection environment on the influence of chance, adaptation and history effects, we considered a combined model that includes the contribution of the selection environment to fitness change (figure 4). We found that environment was a significant contributor to variation in fitness change of the evolved populations. The effect of deep history remained significant whereas shallow history did not.

### (e) Influence of phylogeny on adaptation

It is possible that the phylogenetic relationships among strains predict the influence of deep history on evolutionary outcomes. Alternatively, the effect may be idiosyncratic, such that the effect of deep history varies unpredictably over the phylogeny. To test for a role of phylogenetic signal in determining responses we measured the influence of phylogenetic relationships inferred considering either the core or accessory genomes of progenitor strains on degree of fitness change. We also considered the predictive value of a dendrogram based on growth of progenitor strains in 94 distinct environments (Material and Methods, [65]). The null hypothesis in these tests is that a phenotypic trait will be distributed randomly over a phylogeny or dendrogram, that is, without phylogenetic signal. This hypothesis was rejected in only three instances (of 48 possible, given by four environments, four shallow histories and three phylogeny/dendrogram combinations). Notwithstanding the low statistical power of individual tests, this overall lack of support for any phylogenetic signal is consistent with fitness change not being predictable on the basis of either the phylogenetic relationship or initial ecological performance of progenitor strains (electronic supplementary material, table S3).

## 4. Discussion

We quantified the influence of chance, history, adaptation and environment on evolutionary outcomes by analysing evolved fitness changes of a series of populations founded from divergent natural isolate strains of *E. coli*. We found that differences in deep history were consistently reflected in differences in fitness change and final fitness. By contrast, introduced mutations representing shallow historical differences had little effect on the final fitness of populations but did affect the pattern of fitness change. These findings were consistent across four tested evolution environments, though environment did affect the relative success of strains. Evidently, evolutionary outcomes were strongly dependent on combinations of history and environmental factors.

Several laboratory evolution experiments have used controlled and replicated treatments to measure the influence of chance and genetic history on evolutionary outcomes [37,42,43,46,51,66–68]. For example, Travisano *et al*., [43] found that a period of selection in glucose caused fitness differences between populations that were reduced, but not erased, following a subsequent period of selection in maltose. Similar studies have found differences in fitness change between populations with histories of selection in different environments, including selection for antibiotic resistance mutations [14,36,40]. However, these studies have generally considered the effect of history between very closely related strains. By contrast, the four natural isolate strains that represented the deep history of our experimental design were very divergent, sharing as few as 57% of their genes (strains E704 and FBGM17 share 3485 genes as their core genome but have a combined 6059 genes in their pan genome) [52]. This divergence was reflected in large initial fitness differences between the founder strains and underlies deep history as the most important determinant of the final fitness reached by evolved populations (figure 2).

Deep history was also a significant contributor to differences in changes in fitness occurring during our experiment. It is notable, however, that shallow history explained as much variation in fitness change as deep history in the glucose environment and interacted with deep history in two other environments (figure 3). While the importance of shallow history in glucose may, in part, be due to the added mutations being originally selected for their fitness effects in that environment, its contribution to fitness change in other environments underlines how sensitive evolutionary outcomes can be to even small differences in the starting genotypes of populations. Given this interpretation, it is perhaps surprising that inevitable chance differences in the mutations substituted in replicate populations did not generally cause chance to be more influential in our experiment. One explanation is that mutations arising early in the adaptation of replicate populations to novel environments targeted similar pathways, limiting the opportunity for divergent interactions with subsequent mutations. If our experiment had been continued for longer we expect that chance would become increasingly influential relative to shallow history as genetic differences between replicates become large relative to those initially introduced.

There are two broad and non-exclusive explanations for how history might affect fitness increases occurring in our experiment. The effect and identity of at least some available beneficial mutations might be idiosyncratic, depending on the specific genotype defining different strains [11,69–71]. In essence, starting populations are at places on the fitness landscape with different available mutational paths to fitter phenotypes, creating phylogenetic inertia or constraint. Alternatively, different outcomes might depend on the effect of new mutations depending on some global phenotypic property of a strain, a mechanism known as macroscopic epistasis [72]. This possibility is supported by several studies finding that a significant portion of mutation effects can be explained by the starting fitness of a recipient strain independent of its specific genotype, following a pattern known as diminishing-returns epistasis [24–26,41,73,74].

We find evidence consistent with both explanations presented above, depending on the level of divergence between founding strains. Considering all 16 of the founder strains evolved in each environment, we found no clear effect of initial fitness on fitness change (electronic supplementary material, figure S2). This absence of overall pattern, however, hid a significant effect of decreasing returns at the level of shallow history. In all but one case (all but derivatives of B175 in the acetate environment) derivatives of a progenitor natural isolate strain with higher initial fitness achieved smaller subsequent fitness increases (electronic supplementary material, table S2). This pattern is consistent with the mechanism of diminishing-returns epistasis, though we note that the same pattern might occur through specific mutation interactions if antagonistic interactions become systematically more likely in higher fitness genotypes [69–71]. In any case, the different effect of initial fitness on subsequent fitness changes seen at the two levels of history indicates that opportunities for fitness improvement are not consistent across different levels of strain divergence, even during adaptation to the same environment.

A possible explanation for differences in the pattern of diminishing returns at deep and shallow levels of history is that it depends on the distance of starting populations to a fitness peak [69,75,76]. These distances vary much more at the level of deep compared to shallow history. This effect, however, predicts higher fitness increases in populations founded by low fitness natural isolate strains and is, therefore, expected to reinforce an overall pattern of diminishing returns, which was not seen. An alternative possibility is that populations started by low fitness natural isolate strains were far enough from fitness peaks that many beneficial mutations could occur and, because populations were asexual, interfere with each other's fixation [77]. This interference can impose a ‘speed limit’ to adaptation, but again, does not explain lower fitness populations having smaller fitness increases. Whatever the explanation for different levels of history having different influence on diminishing returns it has evolutionary consequences, implying that predictability of evolutionary responses might be limited to closely related genotypes.

Finally, we emphasize that our focus here was on factors affecting changes in the fitness of evolving populations. Of course, similar fitness outcomes can result from distinct underlying phenotypic changes caused by different genetic changes [78]. This many-to-one mapping means that the influence of the factors we consider might differ when assessed at different biological levels, e.g. changes in genotype or in lower-level phenotypes such as the specific metabolic and physiological changes underlying fitness improvement. Specifically, evolutionary outcomes assessed at the level of fitness are likely to be more convergent, and less contingent, than outcomes assessed using lower-level phenotypes or genotypes [26,37,79]. For this reason, our finding of consistently significant effects of history is likely to be conservative with respect to its effects acting at lower levels of biological organization.

In summary, we find that adaptation to the selective environment, deep genetic history and distinct shallow histories can all affect the evolution of a population. A consequence of history contributing to evolutionary outcomes, especially as it interacts with environment, is that evolution will often be unpredictable. Evidently, even small genetic differences between populations can change either the availability or benefit of specific evolutionary paths [80].

## Data Availability

Data and analysis scripts are available from the Dryad Digital Repository (https://doi.org/10.5061/dryad.4f4qrfjfs) [81]. Electronic supplementary material is available online [82].

## References

[1] Blount ZD, Lenski RE, Losos JB. 2018 Contingency and determinism in evolution: replaying life's tape. Science **362**, eaam5979 12. (10.1126/science.aam5979) 30409860

[2] Wright S. 1932 The roles of mutation, inbreeding, crossbreeding and selection in evolution. *Proceedings of the sixth international congress of genetics* Vol. 1, 356-366.

[3] Sailer ZR, Harms MJ. 2017 High-order epistasis shapes evolutionary trajectories. PLoS Comput. Biol. **13**, e1005541 16. (10.1371/journal.pcbi.1005541) 28505183 PMC5448810

[4] Poelwijk FJ, Kiviet DJ, Weinreich DM, Tans SJ. 2007 Empirical fitness landscapes reveal accessible evolutionary paths. Nature **445**, 383-386. (10.1038/nature05451) 17251971

[5] Szendro IG, Franke J, Visser JAGMD, Krug J. 2013 Predictability of evolution depends nonmonotonically on population size. Proc. Natl Acad. Sci. USA **110**, 571-576. (10.1073/pnas.1213613110) 23267075 PMC3545761

[6] Rozen DE, Habets MGJL, Handel A, Visser JAGMD. 2008 Heterogeneous adaptive trajectories of small populations on complex fitness landscapes. PLoS ONE **3**, e1715. (10.1371/journal.pone.0001715) 18320036 PMC2248617

[7] Silander O, Tenaillon O, Chao L. 2007 Understanding the evolutionary fate of finite populations: the dynamics of mutational effects. PLoS Biol. **5**, e94. (10.1371/journal.pbio.0050094) 17407380 PMC1845161

[8] Schenk MF, Szendro IG, Krug J, Visser JAGMD. 2012 Quantifying the adaptive potential of an antibiotic resistance enzyme. PLoS Genet. **8**, e1002783. (10.1371/journal.pgen.1002783) 22761587 PMC3386231

[9] Lenormand T, Chevin L-M, Bataillon T. 2016 Parallel evolution: what does it (not) tell us and why is it (still) interesting? In Chance in evolution (eds CH Pence, G Ramsey). Chicago, IL: Chicago University Press.

[10] Blount ZD, Borland CZ, Lenski RE. 2008 Historical contingency and the evolution of a key innovation in an experimental population of *Escherichia coli*. Proc. Natl Acad. Sci. **105**, 7899-7906. (10.1073/pnas.0803151105) 18524956 PMC2430337

[11] Woods RJ, Barrick JE, Cooper TF, Shrestha U, Kauth MR, Lenski RE. 2011 Second-order selection for evolvability in a large *Escherichia coli* population. Science **331**, 1433-1436. (10.1126/science.1198914) 21415350 PMC3176658

[12] Tenaillon O, Rodríguez-Verdugo A, Gaut RL, McDonald P, Bennett AF, Long AD, Gaut BS. 2012 The molecular diversity of adaptive convergence. Science **335**, 457-461. (10.1126/science.1212986) 22282810

[13] MacLean R, Bell G. 2003 Divergent evolution during an experimental adaptive radiation. Proc. Biol. Sci. **270**, 1645-1650. 12908987 10.1098/rspb.2003.2408 PMC1691418

[14] Gifford DR, Toll-Riera M, MacLean RC. 2016 Epistatic interactions between ancestral genotype and beneficial mutations shape evolvability in *Pseudomonas aeruginosa*. Evolution **70**, 1659-1666. (10.1111/evo.12958) 27230588

[15] Salverda MLM, Dellus E, Gorter FA, Debets AJM, Oost J van der, Hoekstra RF, Tawfik DS, Visser JAGMD. 2011 Initial mutations direct alternative pathways of protein evolution. PLoS Genet. **7**, e1001321. (10.1371/journal.pgen.1001321) 21408208 PMC3048372

[16] Conway Morris S. 2010 Evolution: like any other science it is predictable. Phil. Trans. R. Soc. B **365**, 133-145. (10.1098/rstb.2009.0154) 20008391 PMC2842699

[17] Gallie J, Bertels F, Remigi P, Ferguson GC, Nestmann S, Rainey PB. 2019 Repeated phenotypic evolution by different genetic routes in *Pseudomonas fluorescens* SBW25. Mol. Biol. Evol. **36**, msz040. (10.1093/molbev/msz040) PMC6519391 30835268

[18] Trontelj P, Blejec A, Fišer C. 2012 Ecomorphological convergence of cave communities. Evolution **66**, 3852-3865. (10.1111/j.1558-5646.2012.01734.x) 23206142

[19] Land MF, Fernald RD. 1992 The evolution of eyes. Annu. Rev. Neurosci. **15**, 1-29. (10.1146/annurev.ne.15.030192.000245) 1575438

[20] Gilding EK et al*.* 2020 Neurotoxic peptides from the venom of the giant Australian stinging tree. Sci. Adv. **6**, eabb8828. (10.1126/sciadv.abb8828) 32938666 PMC7494335

[21] Gallant JR et al. 2014 Genomic basis for the convergent evolution of electric organs. Science **344**, 1522-1525. (10.1126/science.1254432) 24970089 PMC5541775

[22] Sailer ZR, Harms MJ. 2017 Molecular ensembles make evolution unpredictable. Proc. Natl Acad. Sci. USA **114**, 11 938-11 943. (10.1073/pnas.1711927114) 29078365 PMC5691298

[23] Peng F, Widmann S, Wünsche A, Duan K, Donovan KA, Dobson RCJ, Lenski RE, Cooper TF. 2018 Effects of beneficial mutations in *pykF* gene vary over time and across replicate populations in a long-term experiment with bacteria. Mol. Biol. Evol. **35**, 202-210. (10.1093/molbev/msx279) 29069429 PMC5850340

[24] Khan AI, Dinh DM, Schneider D, Lenski RE, Cooper TF. 2011 Negative epistasis between beneficial mutations in an evolving bacterial population. Science **332**, 1193-1196. (10.1126/science.1203801) 21636772

[25] Wünsche A, Dinh DM, Satterwhite RS, Arenas CD, Stoebel DM, Cooper TF. 2017 Diminishing-returns epistasis decreases adaptability along an evolutionary trajectory. Nat. Ecol. Evol. **1**, s41559-016-0061. (10.1038/s41559-016-0061) 28812657

[26] Kryazhimskiy S, Rice DP, Jerison ER, Desai MM. 2014 Global epistasis makes adaptation predictable despite sequence-level stochasticity. Science **344**, 1519-1522. (10.1126/science.1250939) 24970088 PMC4314286

[27] Losos JB, Jackman TR, Larson A, Querioz K, Rodriguez-Schettino L.1998 Contingency and determinism in replicated adaptive radiations of island lizards. Science **279**, 2115-2118. (10.1126/science.279.5359.2115) 9516114

[28] Kolbe JJ, Leal M, Schoener TW, Spiller DA, Losos JB. 2012 Founder effects persist despite adaptive differentiation: a field experiment with lizards. Science **335**, 1086-1089. (10.1126/science.1209566) 22300849

[29] Wiser MJ, Ribeck N, Lenski RE. 2013 Long-term dynamics of adaptation in asexual populations. Science **342**, 1364-1367. (10.1126/science.1243357) 24231808

[30] Lenski RE et al. 2015 Sustained fitness gains and variability in fitness trajectories in the long-term evolution experiment with *Escherichia coli*. Proc. R. Soc. B **282**, 20152292. (10.1098/rspb.2015.2292) PMC4707762 26674951

[31] Quandt EM, Deatherage DE, Ellington AD, Georgiou G, Barrick JE. 2014 Recursive genomewide recombination and sequencing reveals a key refinement step in the evolution of a metabolic innovation in *Escherichia coli*. Proc. Natl Acad. Sci. USA **111**, 2217-2222. (10.1073/pnas.1314561111) 24379390 PMC3926077

[32] Quandt EM, Gollihar J, Blount ZD, Ellington AD, Georgiou G, Barrick JE. 2015 Fine-tuning citrate synthase flux potentiates and refines metabolic innovation in the Lenski evolution experiment. Elife **4**, e09696. (10.7554/elife.09696) 26465114 PMC4718724

[33] Cooper TF, Lenski RE. 2010 Experimental evolution with *E. coli* in diverse resource environments. I. Fluctuating environments promote divergence of replicate populations. BMC Evol. Biol. **10**, 11. (10.1186/1471-2148-10-11) 20070898 PMC2827396

[34] Meyer JR, Dobias DT, Weitz JS, Barrick JE, Quick RT, Lenski RE. 2012 Repeatability and contingency in the evolution of a key innovation in phage lambda. Science **335**, 428-432. (10.1126/science.1214449) 22282803 PMC3306806

[35] Hekstra DR, Leibler S. 2012 Contingency and statistical laws in replicate microbial closed ecosystems. Cell **149**, 1164-1173. (10.1016/j.cell.2012.03.040) 22632978

[36] Phillips KN, Castillo G, Wünsche A, Cooper TF. 2016 Adaptation of *Escherichia coli* to glucose promotes evolvability in lactose. Evolution **70**, 465-470. (10.1111/evo.12849) 26748670

[37] Melnyk AH, Kassen R. 2011 Adaptive landscapes in evolving populations of *Pseudomonas fluorescens*. Evolution **65**, 3048-3059. (10.1111/j.1558-5646.2011.01333.x) 22023573

[38] Gloria-Soria A, Mendiola SY, Morley VJ, Alto BW, Turner PE. 2020 Prior evolution in stochastic versus constant temperatures affects RNA virus evolvability at a thermal extreme. Ecol. Evol. **10**, 5440-5450. (10.1002/ece3.6287) 32607165 PMC7319105

[39] Card KJ, LaBar T, Gomez JB, Lenski RE. 2019 Historical contingency in the evolution of antibiotic resistance after decades of relaxed selection. PLoS Biol. **17**, e3000397. (10.1371/journal.pbio.3000397) 31644535 PMC6827916

[40] Burch CL, Chao L. 2000 Evolvability of an RNA virus is determined by its mutational neighbourhood. Nature **406**, 625-628. (10.1038/35020564) 10949302

[41] Barrick JE, Kauth MR, Strelioff CC, Lenski RE. 2010 *Escherichia coli rpoB* mutants have increased evolvability in proportion to their fitness defects. Mol. Biol. Evol. **27**, 1338-1347. (10.1093/molbev/msq024) 20106907 PMC2872623

[42] Rebolleda-Gómez M, Travisano M. 2019 Adaptation, chance, and history in experimental evolution reversals to unicellularity. Evolution **73**, 73-83. (10.1111/evo.13654) 30520011 PMC6590667

[43] Travisano M, Mongold J, Bennett A, Lenski R. 1995 Experimental tests of the roles of adaptation, chance, and history in evolution. Science **267**, 87-90. 7809610 10.1126/science.7809610

[44] Dickinson BC, Leconte AM, Allen B. 2013 Experimental interrogation of the path dependence and stochasticity of protein evolution using phage-assisted continuous evolution. Proc. Natl Acad. Sci. USA **110**, 9007-9012. (10.1073/pnas.1220670110/) 23674678 PMC3670371

[45] Couce A, Tenaillon OA. 2015 The rule of declining adaptability in microbial evolution experiments. Front. Genet. **6**, 99. (10.3389/fgene.2015.00099) 25815007 PMC4356158

[46] Moore F, Woods R. 2006 Tempo and constraint of adaptive evolution in *Escherichia coli* (Enterobacteriaceae, Enterobacteriales). Biol. J. Linnean Soc. **88**, 403-411. (10.1111/j.1095-8312.2006.00629.x)

[47] Hall AR, Griffiths VF, MacLean RC, Colegrave N. 2010 Mutational neighbourhood and mutation supply rate constrain adaptation in *Pseudomonas aeruginosa*. Proc. R. Soc. B **277**, 643-650. (10.1098/rspb.2009.1630) PMC2842691 19889704

[48] Palmer AC, Toprak E, Baym M, Kim S, Veres A, Bershtein S, Kishony R. 2017 Delayed commitment to evolutionary fate in antibiotic resistance fitness landscapes. Nat. Commun. **6**, 1-8. (10.1038/ncomms8385) PMC4548896 26060115

[49] Ogbunugafor CB, Hartl D. 2016 A pivot mutation impedes reverse evolution across an adaptive landscape for drug resistance in *Plasmodium vivax*. Malaria J. **15**, 1-10. (10.1186/s12936-016-1090-3) PMC4727274 26809718

[50] Simões P, Santos J, Fragata I, Mueller LD, Rose MR, Matos M. 2008 How repeatable is adaptive evolution? The role of geographical origin and founder effects in laboratory adaptation. Evolution **62**, 1817-1829. (10.1111/j.1558-5646.2008.00423.x) 18489721

[51] Lenski RE, Rose MR, Simpson SC, Tadler SC. 1991 Long-term experimental evolution in *Escherichia coli*. I. Adaptation and divergence during 2,000 generations. Am. Nat. **138**, 1315-1341.

[52] Wang Y et al. 2016 Benefit of transferred mutations is better predicted by the fitness of recipients than by their ecological or genetic relatedness. Proc. Natl Acad. Sci. USA **113**, 5047-5052. (10.1073/pnas.1524988113) 27091964 PMC4983819

[53] Cooper TF, Rozen DE, Lenski RE. 2003 Parallel changes in gene expression after 20,000 generations of evolution in *Escherichia coli*. Proc. Natl Acad. Sci. USA **100**, 1072-1077. (10.1073/pnas.0334340100) 12538876 PMC298728

[54] Hall AE, Karkare K, Cooper VS, Bank C, Cooper TF, Moore FB-G. 2019 Environment changes epistasis to alter trade-offs along alternative evolutionary paths. Evolution **73**, 2094-2105. (10.1111/evo.13825) 31418459

[55] Mey MD, Maeseneire SD, Soetaert W, Vandamme E. 2007 Minimizing acetate formation in *E. coli* fermentations. J. Ind. Microbiol. Biot. **34**, 689-700. (10.1007/s10295-007-0244-2) 17668256

[56] Ram Y, Dellus-Gur E, Bibi M, Karkare K, Obolski U, Feldman MW, Cooper TF, Berman J, Hadany L. 2019 Predicting microbial growth in a mixed culture from growth curve data. Proc. Natl Acad. Sci. USA **116**, 14 698-14 707. (10.1073/pnas.1902217116) 31253703 PMC6642348

[57] Laing C, Buchanan C, Taboada EN, Zhang Y, Kropinski A, Villegas A, Thomas JE, Gannon VP. 2010 Pan-genome sequence analysis using Panseq: an online tool for the rapid analysis of core and accessory genomic regions. BMC Bioinf. **11**, 461. (10.1186/1471-2105-11-461) PMC2949892 20843356

[58] Felsenstein J. 1989 PHYLIP - Phylogeny Inference Package (Version 3.2). Cladistics **5**, 164-166.

[59] Seemann T. 2014 Prokka: rapid prokaryotic genome annotation. Bioinformatics **30**, 2068-2069. (10.1093/bioinformatics/btu153) 24642063

[60] Page AJ et al. 2015 Roary: rapid large-scale prokaryote pan genome analysis. Bioinformatics **31**, 3691-3693. (10.1093/bioinformatics/btv421) 26198102 PMC4817141

[61] R core team. 2021 R: a language and environment for statistical computing. https://www.R-project.org/.

[62] Bates D, Mächler M, Bolker B, Walker S. 2015 Fitting linear mixed-effects models using lme4. J. Stat. Softw. **67**, 1-48. (10.18637/jss.v067.i01)

[63] Wang Y, Arenas CD, Stoebel DM, Cooper TF. 2013 Genetic background affects epistatic interactions between two beneficial mutations. Biol. Lett. **9**, 20120328. (10.1098/rsbl.2012.0328) 22896270 PMC3565476

[64] Chou HH, Chiu HC, Delaney NF, Segre D, Marx CJ. 2011 Diminishing returns epistasis among beneficial mutations decelerates adaptation. Science **332**, 1190-1192. (10.1126/science.1203799) 21636771 PMC3244271

[65] Flynn KM, Cooper TF, Moore FB-G, Cooper VS. 2013 The environment affects epistatic interactions to alter the topology of an empirical fitness landscape. PLoS Genet. **9**, e1003426. (10.1371/journal.pgen.1003426) 23593024 PMC3616912

[66] Lenski RE, Travisano M. 1994 Dynamics of adaptation and diversification: a 10,000-generation experiment with bacterial populations. Proc. Natl Acad. Sci. USA **91**, 6808-6814. 8041701 10.1073/pnas.91.15.6808 PMC44287

[67] Jasmin J, Zeyl C. 2012 Life-history evolution and density-dependent growth in experimental populations of yeast. Evolution **66**, 3789-3802. (10.1111/j.1558-5646.2012.01711.x) 23206137

[68] Maharjan RP, Ferenci T. 2013 Epistatic interactions determine the mutational pathways and coexistence of lineages in clonal *Escherichia coli* populations. Evolution **67**, 2762-2768. (10.1111/evo.12137) 24033182

[69] Lyons DM, Zou Z, Xu H, Zhang J. 2020 Idiosyncratic epistasis creates universals in mutational effects and evolutionary trajectories. Nat. Ecol. Evol. **4**, 1685-1693. (10.1038/s41559-020-01286-y) 32895516 PMC7710555

[70] Kryazhimskiy S. 2021 Emergence and propagation of epistasis in metabolic networks. Elife **10**, e60200. (10.7554/elife.60200) 33527897 PMC7924954

[71] Bakerlee CW, Ba ANN, Shulgina Y, Echenique JIR, Desai MM. 2022 Idiosyncratic epistasis leads to global fitness-correlated trends. Science **376**, 630-635. (10.1126/science.abm4774) 35511982 PMC10124986

[72] Good BH, Desai MM. 2015 The impact of macroscopic epistasis on long-term evolutionary dynamics. Genetics **199**, 177-190. (10.1534/genetics.114.172460) 25395665 PMC4286683

[73] Perfeito L, Sousa A, Bataillon T, Gordo I. 2014 Rates of fitness decline and rebound suggest pervasive epistasis. Evolution **68**, 150-162. (10.1111/evo.12234) 24372601 PMC3912910

[74] Szamecz B et al. 2014 The genomic landscape of compensatory evolution. PLoS Biol. **12**, e1001935. (10.1371/journal.pbio.1001935) 25157590 PMC4144845

[75] Blanquart F, Achaz G, Bataillon T, Tenaillon O. 2014 Properties of selected mutations and genotypic landscapes under Fisher's geometric model. Evolution **68**, 3537-3554. (10.1111/evo.12545) 25311558 PMC4326662

[76] Greene D, Crona K. 2014 The changing geometry of a fitness landscape along an adaptive walk. PLoS Comput. Biol. **10**, e1003520. (10.1371/journal.pcbi.1003520) 24853069 PMC4031059

[77] Gerrish P, Lenski R. 1998 The fate of competing beneficial mutations in an asexual population. Genetica **102–103**, 127-144. (10.1023/a:1017067816551) 9720276

[78] Schluter D, Clifford EA, Nemethy M, McKinnon JS. 2004 Parallel evolution and inheritance of quantitative traits. Am. Nat. **163**, 809-822. (10.1086/383621) 15266380

[79] Bedhomme S, Lafforgue G, Elena SF. 2013 Genotypic but not phenotypic historical contingency revealed by viral experimental evolution. BMC Evol. Biol. **13**, 1. (10.1186/1471-2148-13-46) 23421472 PMC3598485

[80] Crona K, Luo M, Greene D. 2020 An uncertainty law for microbial evolution. J. Theor. Biol. **489**, 110155. (10.1016/j.jtbi.2020.110155) 31926205

[81] Smith CE, Smith ANH, Cooper TF, Moore FB-G. 2022 Data from: Fitness of evolving bacterial populations is contingent on deep and shallow history but only shallow history creates predictable patterns. Dryad Digital Repository. (10.5061/dryad.4f4qrfjfs) PMC9470251 36100026

[82] Smith CE, Smith ANH, Cooper TF, Moore FB-G. 2022 Fitness of evolving bacterial populations is contingent on deep and shallow history but only shallow history creates predictable patterns. *Figshare*. (10.6084/m9.figshare.c.6179392) PMC9470251 36100026

